# Supplementary material for: The Response Regulator VC1795 of Vibrio Pathogenicity Island-2 Contributes to Intestinal Colonization by Vibrio cholerae
Source: Int J Mol Sci. 2023 Aug 31;24(17):13523. doi: 10.3390/ijms241713523 (PMC10487451; doi:10.3390/ijms241713523)
Supplement: Supplementary file 1 [file ijms-24-13523-s001.zip › ijms-2552480-supplementary.pdf]

Fig. S1

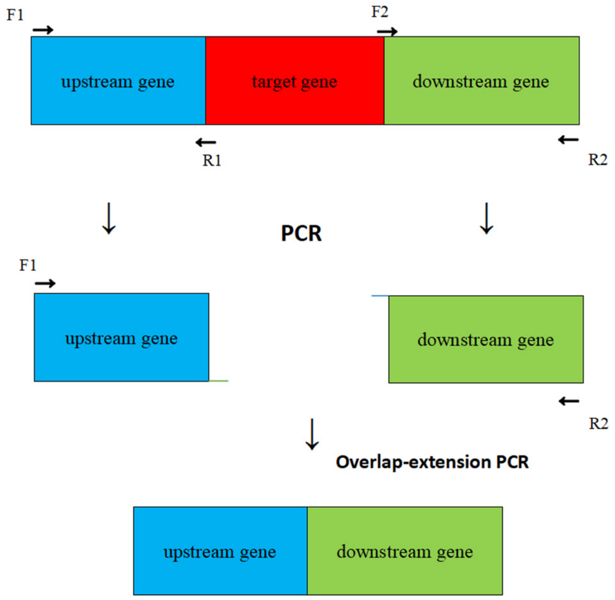

Fig. S1 Graphical process of the deletion of the target gene in this study.

Fig. S2

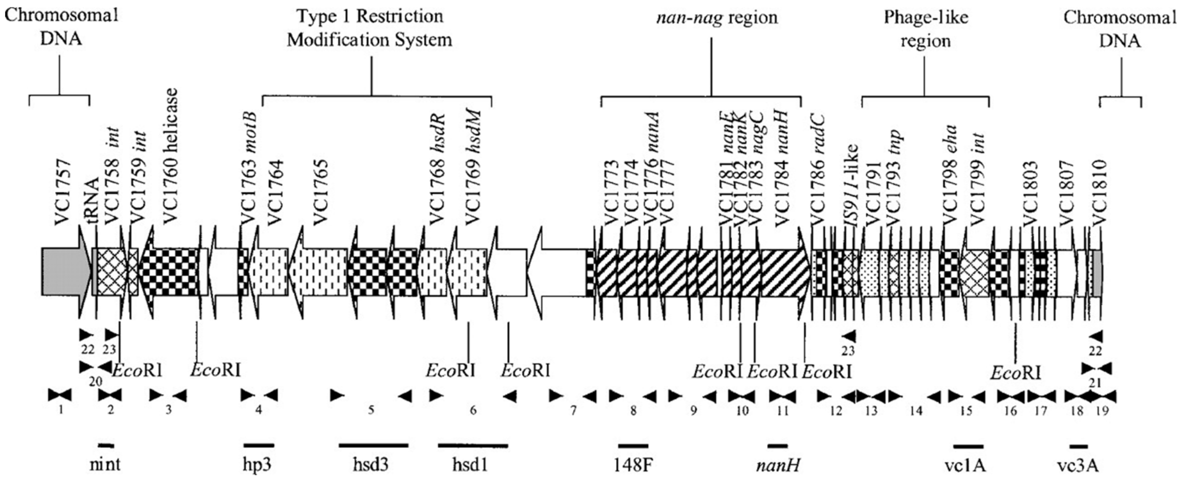

Fig. S2 Schematic representation and genetic organization of the 57·3 kb VPI-2 on the *V. cholerae* N16961 genome [1].

[1] Jermyn WS, Boyd EF. Characterization of a novel *Vibrio* pathogenicity island (VPI-2) encoding neuraminidase (*nanH*) among toxigenic *Vibrio cholerae* isolates.

Microbiology (Reading). 2002 Nov;148(Pt 11):3681-3693.

Fig. S3

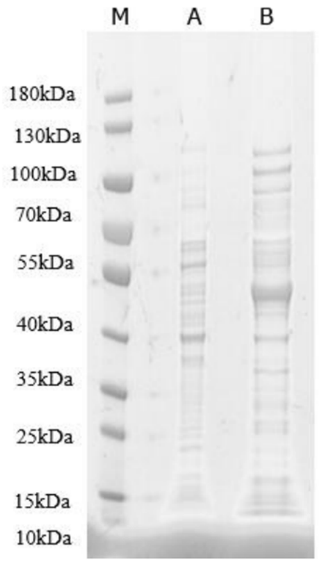

Fig. S3 The results of *VC1795* DNA pull-down assay by SDS-PAGE. M, 10~180kDa protein marker. A, the eluted protein of *VC1795* DNA pull-down. B, bacterial lysis of the whole protein.

Fig. S4

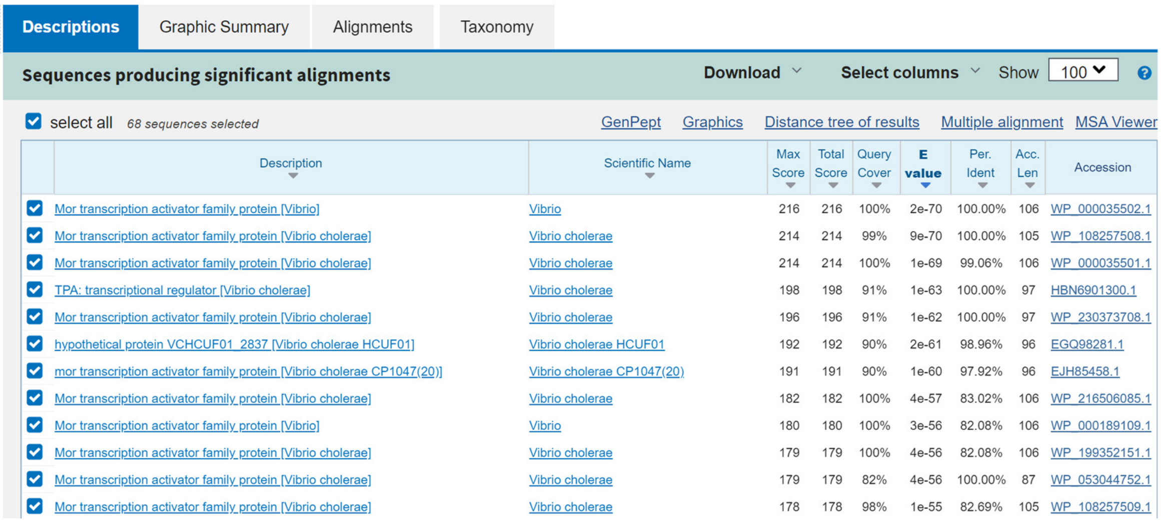

Descriptions | Graphic Summary | Alignments | Taxonomy

Sequences producing significant alignments — Download — Select columns — Show 100

select all 68 sequences selected — GenPept Graphics Distance tree of results Multiple alignment MSA Viewer

Description	Scientific Name	Max Score	Total Score	Query Cover	E value	Per. Ident	Acc. Len	Accession
Mor transcription activator family protein [Vibrio]	Vibrio	216	216	100%	2e-70	100.00%	106	WP_000035502.1
Mor transcription activator family protein [Vibrio cholerae]	Vibrio cholerae	214	214	99%	9e-70	100.00%	105	WP_108257508.1
Mor transcription activator family protein [Vibrio cholerae]	Vibrio cholerae	214	214	100%	1e-69	99.06%	106	WP_000035501.1
TPA: transcriptional regulator [Vibrio cholerae]	Vibrio cholerae	198	198	91%	1e-63	100.00%	97	HBN6901300.1
Mor transcription activator family protein [Vibrio cholerae]	Vibrio cholerae	196	196	91%	1e-62	100.00%	97	WP_230373708.1
hypothetical protein VCHCUF01_2837 [Vibrio cholerae HCUF01]	Vibrio cholerae HCUF01	192	192	90%	2e-61	98.96%	96	EGQ98281.1
mor transcription activator family protein [Vibrio cholerae CP1047(20)]	Vibrio cholerae CP1047(20)	191	191	90%	1e-60	97.92%	96	EJH85458.1
Mor transcription activator family protein [Vibrio cholerae]	Vibrio cholerae	182	182	100%	4e-57	83.02%	106	WP_216506085.1
Mor transcription activator family protein [Vibrio]	Vibrio	180	180	100%	3e-56	82.08%	106	WP_000189109.1
Mor transcription activator family protein [Vibrio cholerae]	Vibrio cholerae	179	179	100%	4e-56	82.08%	106	WP_199352151.1
Mor transcription activator family protein [Vibrio cholerae]	Vibrio cholerae	179	179	82%	4e-56	100.00%	87	WP_053044752.1
Mor transcription activator family protein [Vibrio cholerae]	Vibrio cholerae	178	178	98%	1e-55	82.69%	105	WP_108257509.1

Fig. S4 The alignment results of VC1795 amino acid sequence in National Center for Biotechnology Information (NCBI).

Fig. S5

CCGCCTTCAAACAAGCCAATCTTTAAATGCCTTTTCATTAGAAGAGGCATTT
CTTTCTGAAACGCCCTCAAATCAGTTTCTAGCCGTGATGCGAGTATTCGCTT
GTCTAAAAAAGTTTAAATCAATCTGGTGCGATTTAAACAGGGTTTAAACAT
GGTTTCTGCACATATCAGTGCTTATATATAACTCCCACAATGCCCAGTCCCCT
TTATTAACAGCAACTCCTGTTAATTTAGTTATTACTGCAACGGGGATTATTCT
TGTATTGCTGTTGGGGCATATGGAGATAAGTCGGTTCTCCATTAAATGGCAA
TACATTTGCCATCAACTTAGCGTAGCCCTATCTGCAAATTTGGGTAGGGTTA
CGCTAGGTTCTTCGATGTCTATATAGAGGATAACG

Fig. S5 Putative CRP binding sites (red and underlined) at the *VC1795* promoter region.

Table S1 Bacterial strains and plasmids used in this study

Strains or plasmids	Genotype or relevant characteristics	Source
Vibrio cholerae Strains		
E12382	Virulent strain, O1 El Tor type; PmB[r]	Lab collection
Δ*VC1795*	E12382, deletion of*VC1795*; PmB[r]	This study
Δ*VC1795*::p*VC1795*	Δ*VC1795* containing plasmid p*VC1795*; PmB[r]	This study
Δ*crp*	E12382, deletion of *crp*; PmB[r]	This study
Δ*cytR*	E12382, deletion of *cytR*; PmB[r]	This study
Δ*VC1807*	E12382, deletion of *VC1807*; PmB[r]	This study
Δ*VC1794*	E12382, deletion of*VC1794*; PmB[r]	This study
Escherichia coli Strains		
S17-1(λpir)	*Tpr Smr recA thi pro rK- mK- RP4:2-Tc:Mu Km Tn7 λ pir* (*thi pro hsdR hsdM+ recA RP4-2-Tc : :*	Lab collection

BL21(DE3)	Host strain for protein expression	Lab collection
BL21/pET-*VC1795*	BL21(DE3) with pET28a carrying the *VC1795* gene; Km^r	This study
BL21/pET-*crp*	BL21(DE3) with pET28a carrying the *crp* gene; Km^r	This study
BL21/pET-*cytR*	BL21(DE3) with pET28a carrying the *cytR* gene; Km^r	This study
Plasmids		
pRE112	pGP704 suicide plasmid, *pir* dependent, *oriT*, *oriV*, *sacB*, Cm^r	Lab collection
pBAD33	Arabinose inducible promoter; Cm^r	Lab collection
pET28a	T7 expression vector; Km^r	Lab collection
pET-*VC1795*	pET28a carrying the *VC1795* gene; Km^r	This study
pET-*crp*	pET28a carrying the *crp* gene; Km^r	This study
pET-*cytR*	pET28a carrying the *cytR* gene; Km^r	This study
Δ*VC1795*/pBAD33-*VC1795*-3×FLAG	Δ*VC1795* containing plasmid p*VC1795*-3×FLAG; PmB^r	This study

[a] r = resistant.

Table S2 Primers used in this study

Target genes		Primer sequence (5'-3')
Primers for construction of mutants		
Δ*VC1795*	F	CGAGCTCCGAGTATTCGCTTGTCTAAA
	R	TCCTATCTAGTGATAACTCTCCTTACA
	F	TCATGTAAGGCTAGATAGGAAATTAACCAT
	R	CGGGGTACCTGCCATCGACTAATTGATAC
Δ*crp*	F	CGGGGTACCGTTCAATGGACGACTACCT
	R	GAGACGGGTTAATAATCTCACTTCCTCTGC

	F	GCAGAGGAAGGTGCCCCGATAACCCGTCTC
	R	TCCCCCGGGAAGCATTAGCCAGTGAGG
Δ*cytR*	F	CGAGCTCTTTCGAGCTGAAGCCAATC
	R	CGAAGGTGGGGTATTTTTACCCTCTTTTCTCTATCG
	F	GTAAAAATACCCCACCTTCGAAACCGA
	R	CGGGGTACCTCCGAGGACGACACGATAC
Δ*VC1807*	F	CGAGCTCTTCAGGGATTGAGGAAAGC
	R	GGGGAGCGCTGGACACAGGATAAGGATTCGT
	F	TCCTGTGTCCAGCGCTCCCCTATTTTTTAT
	R	CGGGGTACCGAAGCCTTAGCCAGACGAG
Δ*VC1794*	F	CGAGCTCATGTTAGCTATAGAAAGAGCAATCC
	R	CGACTGACGAGGTTAATTTCCTATCTAGTTATGACGTTTT
	F	GAAATTAACCTCGTCAGTCGATTTATTATCTCAGT
	R	CGGGGTACCTTACCAATAAACTCCACTTGACG
Primers for construction of complemented strain		
Δ*VC1795*::p*VC1795*	F	CGGGGTACCCTTTGATGATCTTGAAAGCGGT
	R	AAAACTGCAGTTATGACGTTTTGAATTTGCGA
Primers for identification of plasmid		
pRE112	F	CACTGTTCGTCCATTTCCG
	R	TTCGTCTCAGCCAATCCCT
pBAD33	F	AACAAAGCGGGACCAAAG
	R	AGAGCGTTCACCGACAAA
pET28a	F	TAATACGACTCACTATAGGG
	R	TGCTAGTTATTGCTCAGCGG

Primers for qRT-PCR

16S rRNA	F	CCCTTATCCTTGTTTGCC
	R	TATCGCTGCCCTCTGTAT
VC1795	F	TCAAAGGTCGCTTGTGATTTC
	R	TTGAGTTCTTCGTGCCACAG
VC1794	F	GCCTCTGAACTTGTGGAACG
	R	TGGCGTACGCTATCTGACAA
VC1803	F	AAATAGCTGGCCGACAACAG
	R	TCCGCTTCTACTCCCTTGTG
VC0575	F	CGTAGGTATGCCCCATGTTC
	R	AAGAAACCCATTGTCCACCA
VC1325	F	GATACCGGCATGTGGGATAC
	R	ATGTCGCCGGACTTAATGAG
VC1442	F	TCTTTCTGGTGCATGGCATA
	R	GCGCTCTTGTCCAAAAAGAC
VC0159	F	GTGGTTGCAACGGCTTTAAT
	R	ACTCGGCGAACAGCTCTTTA
VC1811	F	ATTGCCATTTTGGTGGATGT
	R	CAAAGCCAATCCCTCGTAAA
tcpA	F	TGGGGATTTTCTCATTTCCA
	R	CTTCCTGGTGCAATGGACTT
tcpB	F	TACAAGCGGGAAGCTGAACT
	R	CTGCTGTGAGGCAGTTTTCA
tcpC	F	CTCCGCCTCAGCAAGATTAG

	R	TCGGCATAACCATCATGCTA
tcpD	F	CTGGGAGGTGGAGCCTATTT
	R	AAAAACCAGTTAGCGCTTGGT
tcpE	F	GAGGGAGTGGGCATCTATGA
	R	TGCAGCAATACCACTCGAAA
tcpF	F	CTACGGATTCAAGGGGGAGT
	R	CAGCTAAAAGCCGAGCAGAG
tcpQ	F	GCAGATTGCAGAACAGTCCTC
	R	CCGCATTATCGAACTGAACC
tcpR	F	CCTCATTTATCAAGGCGTCAA
	R	TTCAATCAGTTGTGCATCCAA
tcpS	F	GCACCCGTTTTATCTCCTCA
	R	CGATTACCTTATTGCCCTTGG
tcpT	F	TGCTGTATTTAGCGCGAATG
	R	GTCGCTCCCGATATTTACGA
Primers for EMSA		
VC1795	F	GGTTCTTCGATGTCTATATAGAGGA
	R	GCACTTTGCTTAATGGCTTC
VC1794	F	AACTCACTCCTAGCGCTCAAG
	R	GGTTAATTTCCTATCTAGTTATGACG
4.5S RNA	F	CTGGTCCTCCCGCAACAC

	R	GAGACCCCAGCCACATC
Primers for pull-down		
VC1795	F	AGCACTTTGCTTAATGGCTT
	R	ATGACATTTTTCTTGGTGTCATT
Primers for CHIP		
VC1795	F	CGGAGCTCATGGCACCCACTGATAATCA
	R	GCTCTAGATTACTATTTATCGTCGTCATCTTTGTAGTC<br>GATATCATGATCTTTATAATCACCGTCATGGTCTTTGT<br>AGTCTGACGTTTTGAATTTGCGAA
Primers for protein cloning		
CRP	F	CGCGGATCCATGGTTCTAGGTAAACCTCA
	R	CCGCTCGAGTTAGCGAGTGCCGTAAACCA
CytR	F	CGCGGATCCATGGCGACAATGAAGGATG
	R	CCGCTCGAGTTACTTCTTGCTTGGCGG
VC1795	F	CGCGGATCCATGGCACCCACTGATAATC
	R	CCGCTCGAGATGGTTCTAGGTAAACC

F, forward; R, reverse.

Table S3 Mass spectrometry protein detection results of *VC1795* DNA-pull down

protein	description	score
VC0395	Response regulator	377
Acs	Acetyl-coenzyme A synthetase	304
RpsC	30S ribosomal protein S3	272
SpeA	Biosynthetic arginine decarboxylase	257

MalT	HTH-type transcriptional regulator MalT	246
VC1087	Response regulator	214
GlpB	Anaerobic glycerol-3-phosphate dehydrogenase subunit B	172
PheT	Phenylalanine--tRNA ligase beta subunit	167
HupA	DNA-binding protein	132
CytR	Bacterial regulatory s, lacI family protein	104
AtpD	ATP synthase subunit	103
MenD	2-succinyl-5-enolpyruvyl-6-hydroxy-3-cyclohexene-1-carboxylate synthase	102
Crp	Bacterial regulatory s, crp family protein	101
OmpW	Outer membrane protein W	105
GltA	Citrate synthase	97
YeiP	Elongation factor P-like protein	87
Fur	Ferric uptake regulation protein	85
AcpP	Acyl carrier protein	84
RplQ	50S ribosomal protein L17	83
RplD	50S ribosomal protein L4	82
RpsK	30S ribosomal protein S11	80
PdxH	Pyridoxine/pyridoxamine 5'-phosphate oxidase	74
CutC	Copper homeostasis protein CutC	70
ProQ	RNA chaperone ProQ	69
SecF	Protein-export membrane protein SecF	68
UppS	Ditrans, polycis-undecaprenyl-diphosphate synthase	66
HtpG	Chaperone protein HtpG	65
Gap	Glyceraldehyde-3-phosphate dehydrogenase	60
LysS	Lysine--tRNA ligase	60
MiaB	tRNA-2-methylthio-N(6)-dimethylallyladenosine synthase	58
Gnd	6-phosphogluconate dehydrogenase, decarboxylating	57
PepT	Peptidase T	55
MetK	S-adenosylmethionine synthase	52
Rho	Transcription termination factor Rho	50
HldE	Bifunctional protein HldE	48

VexA	MexH family multidrug efflux RND transporter periplasmic adaptor subunit	47
RplP	50S ribosomal protein L16	46
Lrp	Leucine-responsive regulatory protein	43
KatB	Catalase	41

Table S4 ChIP-seq data of VC1795 transcriptional regulator from *Vibrio cholerae*

peak name	start	end	log2.fold enrichment	gene
output_peak_51a	1940728	1942078	1.39099	*VC1794*
output_peak_47	1709155	1709510	1.38381	*VC1596*
output_peak_52	1947894	1948242	1.38094	*VC1803*
output_peak_26a	674721	676016	1.36418	*VC0633*
output_peak_24b	603519	607217	1.35077	*VC0575*
output_peak_22	516215	516495	1.32396	*deoR*
output_peak_6a	149790	150350	1.30049	*VC0159*
output_peak_40	1409003	1409457	1.26130	*VC1325*
output_peak_54	1953844	1954075	1.22993	*VC1811*
output_peak_44a	1538267	1539120	1.22675	*VC1442*
output_peak_84b	2753915	2754809	1.22675	*rpmJ*
output_peak_103	308442	308929	1.22675	*infC*
output_peak_72a	2345944	2347145	1.22625	*flgB*
output_peak_68	2253835	2254126	1.2234	*gltA*
output_peak_81	2564984	2565228	1.22135	*VC2397*
output_peak_92	2956379	2956838	1.21848	*VC2772*
output_peak_38d	1158288	1160499	1.21753	*VC1091*
output_peak_19a	382590	383274	1.2167	*VC0364*
output_peak_29	678090	678310	1.2167	*VC0637*
output_peak_38c	1158288	1160499	1.21335	*VC1091*
output_peak_21	436048	436287	1.21004	*VC0410*

output_peak_46a	1566851	1567997	1.20888	*VC1457*
output_peak_5	105688	106044	1.20824	*VC0108*
output_peak_49	1730796	1731500	1.20309	*VC1618*
output_peak_56b	1976387	1977680	1.19994	*VC1835*
output_peak_62	2067222	2067503	1.19994	*VC1917*
output_peak_108	689489	689973	1.19968	*glpK*
output_peak_35	1021588	1021850	1.19962	*VC0957*
output_peak_46b	1566851	1567997	1.19958	*VC1456*
output_peak_56a	1976387	1977680	1.19639	*tolB*
output_peak_72c	2345944	2347145	1.19458	*flgD*
output_peak_20	434524	434899	1.18825	*VC0408*
output_peak_95b	61524	62132	1.18825	*VCA0053*
output_peak_97	151093	151468	1.18739	*glpQ*
output_peak_10a	252982	253805	1.18653	*VC0247*
output_peak_28b	677157	677716	1.18653	*VC0635*
output_peak_58	2049913	2050190	1.18653	*nhaB*
output_peak_90a	2926432	2926771	1.18653	VC2751
output_peak_4	75933	76172	1.18468	*VC0076*
output_peak_16	339850	340090	1.18318	*rplL*
output_peak_56c	1976387	1977680	1.18318	*VC1834*
output_peak_34	897179	897465	1.17983	*VC0835*
output_peak_31a	682640	683195	1.17979	*secG*
output_peak_55	1975847	1976119	1.17842	*VC1834*
output_peak_70	2335813	2336038	1.17666	*VC2188*
output_peak_28a	677157	677716	1.17648	*rrmJ*
output_peak_79b	2513760	2514210	1.17648	*VC2357*
output_peak_27	676350	676651	1.17313	*greA*
output_peak_80	2564162	2564394	1.16603	*lpxC*
output_peak_13	266064	266401	1.15972	*VC0260*
output_peak_25a	607507	608289	1.15972	*VC0578*
output_peak_63	2157734	2158112	1.15568	*VC2005*

output_peak_95a	61524	62132	1.15152	*VCA0053*
output_peak_90b	2926432	2926771	1.14929	*VC2750*
output_peak_37	1079204	1079503	1.14553	*VC1009*
output_peak_88	2910002	2910290	1.14022	*VC2738*
